# Analyses of the Differences in Nutritional Quality and Volatile Aroma Compounds in Potato-Based Reconstituted Rice Produced from Different Potato Varieties

**DOI:** 10.3390/foods14213622

**Published:** 2025-10-24

**Authors:** Zongming Guo, Kaifeng Li, Quanfeng Zhang, Fankui Zeng, Huachun Guo

**Affiliations:** 1 Institute of Root and Tuber Crops, College of Agronomy and Biotechnology, Yunnan Agricultural University, Kunming 650201, China; darkskysaber@outlook.com (Z.G.); dtllx04@163.com (K.L.); 2 Food Research and Development Center, Shenzhen University, Shenzhen 518000, China; zquanfeng@szu.edu.cn; 3 Lanzhou Institute of Chemical Physics, Chinese Academy of Sciences, Lanzhou 730030, China; zengfk@licp.cas.cn

**Keywords:** potato-based reconstituted rice, nutritional composition, aroma compounds, fresh potato processing, anthocyanins, carotenoids

## Abstract

Potato-based reconstituted rice represents an innovative staple food solution that addresses nutritional and economic challenges. Using fresh potatoes instead of potato flour eliminates nutrient loss and reduces energy costs associated with traditional processing methods. This study examined reconstituted rice produced from the yellow-fleshed ‘Dianshu 1428’ and purple-fleshed ‘Diancaishu 101’ potato varieties, comparing their nutritional and aromatic profiles with commercial rice-based alternatives. The results demonstrated significant nutritional advantages: potato-based reconstituted rice contains 3 g/100 g dietary fiber, a five-fold higher potassium content, and 11–12 times more iron than conventional rice-based reconstituted rice. Unique aroma compounds, including methional, 2-ethyl-3,5-dimethylpyrazine, α-ionone, and (E,E)-2,6-nonadienal, impart distinctive potato, nutty, fruity, and fatty flavors. Yellow-fleshed varieties contributed 14.1 μg/100 g carotenoids, while purple-fleshed varieties provided 45 mg/100 g anthocyanins. These findings establish that potato-based reconstituted rice offers a superior nutritional composition and unique sensory characteristics compared to traditional alternatives, providing scientific guidance for the selection of the potato variety and product optimization in developing nutritionally enhanced potato staple foods with specific functional and sensory attributes.

## 1. Introduction

Potato is China’s fourth largest food crop, with an annual planting area exceeding 4.7 million hectares and an annual production of over 90 million tons. Both its planting scale and total production rank first globally, accounting for more than one-quarter of the global total [1]. As an important food resource, potatoes are rich in various trace elements such as potassium and calcium, which can effectively compensate for the insufficient intake of trace elements in populations with long-term consumption of the staple foods wheat and rice, as well as refined processed grains [2]. However, currently, potato consumption in China is still dominated by fresh vegetable use (accounting for over 60%) [1], failing to fully utilize its function as a staple food. China has implemented a strategy to promote the transformation of potatoes into a staple food since 2015 by successively developing a series of staple food products such as potato noodles and steamed bread. However, most of these products use potato flour as the raw material, with complex processing procedures, high energy consumption, and high costs, resulting in insufficient market competitiveness of the end products.

Extruded reconstituted rice is a granular rice product like natural rice that is made from starch-based raw materials through homogenization, extrusion, and drying. Also known as “analog rice”, it has already achieved commercial production as a potential staple food substitute. Previous studies mostly used sweet potato, cassava, corn, and various grains as raw materials [3]. They focused on optimizing formulations to enhance the antioxidant properties [4] and resistant starch and dietary fiber contents [5], meeting the nutritional needs of specific populations such as diabetic patients [6] and lactating women [7]. In terms of processing, existing research mainly focuses on optimizing variables such as the extrusion temperature, screw speed, raw material ratio, and post-processing parameters [8]. However, these processes generally rely on dry powder raw materials. Their preparation involves multiple steps, such as material drying and grinding, which not only increase production costs but also cause nutrient loss. In 2023, the Food Research and Development Center of Shenzhen University pioneered the use of high-moisture extrusion technology. This technology directly converts fresh potatoes into 0.5–1 mm rice-like products. It significantly simplifies the processing procedure and reduces storage and transportation losses and production costs, providing a new technical pathway for potato staple food development. The product received a positive market response upon launch due to its innovative and economic advantages.

Eating quality is a key factor affecting consumer acceptance of reconstituted rice, and sensory characteristics are the core of a quality evaluation [9]. Amino acids, proteins, dietary fiber, and bioactive components (such as anthocyanins and carotenoids) in raw materials not only provide functional properties to reconstituted rice but also serve as important material basis for flavor and texture formation [10]. During processing and cooking, these compounds undergo further thermochemical reactions to generate volatile aroma compounds. Existing studies mostly focus on an aroma precursor analysis, sensory evaluation [11], and the effects of cooking methods on eating quality [12], while a systematic identification of volatile compounds remains relatively lacking. It is particularly noteworthy that in traditional reconstituted rice production, the compositional changes after raw materials are dried and ground significantly alter the flavor characteristics of the final product [13]. In contrast, using fresh potatoes as raw materials can better preserve the natural flavors and nutrients, potentially resulting in a richer flavor composition.

Unlike rice, in which 2-acetyl-1-pyrroline and hexanal are the main aroma components, potato volatile compounds are dominated by aldehydes, ketones, and alcohols. Their flavor characteristics are significantly influenced by various factors [14]. For example, key flavor compounds in fried potato chips include aldehydes such as methional, hexanal, and decanal, as well as pyrazine compounds. Purple potatoes produce more prominent Maillard reaction products due to their higher reducing sugar content [15]. However, whether reconstituted rice made from different colored potato varieties differs in the flavor compound composition and overall quality remains unclear. This gap prevents processing companies from easily ensuring flavor consistency in products, creating risks in market acceptance.

Based on this information, this study used two potato varieties independently bred by Yunnan Agricultural University as the raw materials. Using high-moisture extrusion technology, we prepared yellow potato reconstituted rice (YPRR) and purple potato reconstituted rice (PPRR), with rice-based reconstituted rice (RBRR) serving as the control. This study systematically analyzed differences in nutritional components (amino acids and mineral elements). We used headspace solid-phase microextraction gas chromatography–mass spectrometry (HS-SPME-GC-MS) technology to identify volatile flavor compounds. Combined with orthogonal partial least squares discriminant analysis (OPLS-DA), we screened key differential flavor markers. These research results aim to clarify how different raw material varieties affect the flavor quality of novel potato-based reconstituted rice. This study provides a theoretical basis for selecting suitable varieties during processing to stabilize and optimize product flavor.

## 2. Materials and Methods

### 2.1. Materials

Potato tuber materials were produced during winter cropping by the Institute of Root and Tuber Crops of Yunnan Agricultural University in Shuangjiang County, Yunnan Province, China. They were then processed into potato-based reconstituted rice by the Food Research and Development Center of Shenzhen University. Rice-based reconstituted rice (Haihongda Catering Management Co., Ltd., Beijing, China) was purchased from commercial markets.

**Figure 1:**
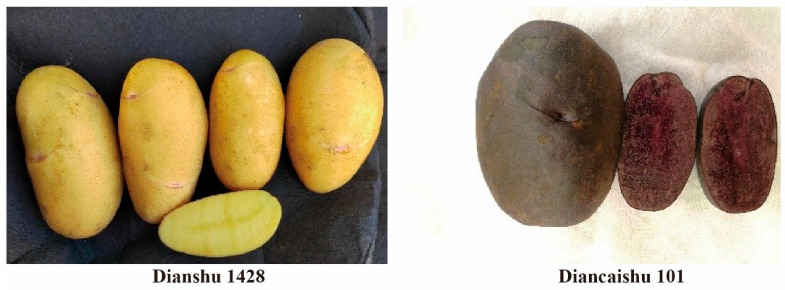
The potato varieties ‘Dianshu 1428’ and ‘Diancaishu 101’ used for reconstituted rice processing.

The main characteristics of ‘Dianshu 1428’ (left panel, Figure 1) include elongated oval-shaped tubers with yellow skin, a deep yellow flesh, and light red eyes. It has a dry matter content of 21.3%, starch content of 17.5%, protein content of 2.15%, and reducing sugar content of 0.20%. It is rich in vitamin C (38.1 mg/100 g), has a good eating quality, and produces a pleasant aroma when cooked. ‘Diancaishu 101’ (right panel, Figure 1) has elongated oval-shaped tubers with purple skin and purple flesh. It has a higher dry matter content of 22.3%, starch content of 16.1%, protein content of 2.39%, reducing sugar content of 0.25%, vitamin C content of 18.89 mg/100 g, and anthocyanin content of 47.5 mg/100 g FW. The nutrient profiles of the above varieties were determined in our laboratory according to Chinese National Standards GB5009.5-2016 [16], GB5009.9-2023 [17], GB5009.3-2016 [18], and GB5009.83-2016 [19].

### 2.2. Determination of the Mineral Element and Amino Acid Contents

Mineral elements were detected according to Chinese National Standard GB5009.268-2016 using inductively coupled plasma mass spectrometry (ICP-MS) [20]. The amino acid content was determined according to National Standard GB5009.124-2016 using an amino acid analyzer [21]. The tests were commissioned to Yunnan CTI Testing Company (Kunming, China), All samples were analyzed before cooking, with three replicates.

### 2.3. Determination of the Main Nutritional Indicators

The protein content was determined according to Chinese National Standard GB5009.5-2016 using the Kjeldahl method [16]. The starch content was measured according to Chinese National Standard GB5009.9-2023 using the acid hydrolysis method [17]. The dry matter content was determined according to Chinese National Standard GB5009.3-2016 using the direct drying method [18]. The reducing sugar content was detected using the direct titration method according to Chinese National Standard GB5009.7-2016 [22]. The β-carotene content was determined using high-performance liquid chromatography according to Chinese National Standard GB5009.83-2016 [19]. The dietary fiber content was measured using high-performance liquid chromatography after enzymatic hydrolysis according to Chinese National Standard GB5009.88-2023 [23]. The total anthocyanin content was determined using the differential pH method according to TZNZ320-2025 [24]. All samples were analyzed before cooking, with three replicates.

### 2.4. Determination of Volatile Flavor Compounds

#### 2.4.1. Sample Cooking and Pretreatment

The three types of rice were cooked using an electric rice cooker (MB-WFS3018Q, Midea Group Co., Ltd., Guangzhou, China) according to Chinese National Standard GB/T 15682-2008 [25]. The ratio of reconstituted rice to pure water was 1:1.5 (V:V). After the rice cooker turned off, the rice was steamed for an additional 20 min. Subsequently, 3 g of each cooked rice sample was weighed and placed into a 25 mL headspace vial. A total of 1 μL of 2-methyl-3-heptanone (0.163 μg/μL, Sigma-Aldrich, St. Louis, MO, USA) was added as an internal standard. After adding 0.3 g of sodium chloride, the sample vial was sealed with a PTFE septum. After equilibration in a water bath at 50 °C for 30 min, solid-phase microextraction was performed using a 75 μm CAR/PDMS fiber for 40 min before detection. The extraction membrane was conditioned at 270 °C for 30 min before use.

#### 2.4.2. GC-MS Parameters

After extraction, the sample was desorbed from the membrane at the GC-MS injection port (Agilent 7890A-5977C, Agilent Technologies, Santa Clara, CA, USA) at 250 °C for 5 min for the gas chromatography–mass spectrometry analysis. The specific analytical conditions were as follows: the column was an HP-5 ms capillary column (30 m × 0.25 mm × 0.25 μm); the carrier gas was helium in splitless injection mode with a flow rate of 1.2 mL·min^−1^. Temperature programming conditions were an initial temperature of 40 °C held for 3 min, an increase to 200 °C at 5 °C·min^−1^, an increase to 230 °C at 10 °C·min^−1^ and a hold for 5 min. The following mass spectrometry conditions were used: electron impact ionization source (EI), electron energy of 70 eV, transfer line temperature of 280 °C, ion source temperature of 230 °C, quadrupole temperature of 150 °C, full scan mode (mass scan range m/z 33-550), and solvent delay of 1 min.

### 2.5. Sensory Evaluation

The three types of reconstituted rice were evaluated after cooking according to the Chinese National Standard GB/T15682-2008 [25] (sensory evaluation of the cooking and eating quality of rice). The evaluation panel consisted of 30 participants, including faculty and doctoral students from the Institute of Tuber Crops. Prior to the sensory evaluation, all participants received relevant training, had experience in evaluating the sensory characteristics of fresh potatoes, and understood the specific requirements for each sensory indicator. The evaluators assessed and scored the reconstituted rice based on five aspects: aroma, stickiness, springiness, hardness, and taste. As the score distribution did not follow a normal distribution, the Kruskal–Wallis test was performed, followed by Dunn’s test for multiple comparisons.

### 2.6. Statistical Analysis

Volatile compounds in reconstituted rice were qualitatively analyzed using ChemStation 1.2.5 software (Agilent Technologies, Santa Clara, CA, USA). Quantification and data analysis methods were the same as those described by Bough et al. [26] and were performed using the internal standard method described in Formula (1). Cluster analysis, partial least squares analysis, and compound correlation analysis were performed using the online analysis platform MetaboAnalyst 6.0 (accessed on 20 October 2025). One-way ANOVA with false discovery rate (FDR) correction was performed using R (4.4.1). Statistical analyses of sensory evaluation results were performed using the packages “agricolae” and “dunn.test”. In the experiment, each reconstituted rice material was analyzed in triplicate, with the data presented as the means ± standard deviations. The odor activity values (OAVs) for key compounds were calculated using Formula (2). The proportion of each characteristic flavor compound was calculated using Formula (3), and the ratio of compounds was represented with the same odor characteristics to the total calculated compounds. The compounds used to calculate the total were the 19 selected key compounds.

(1) $$C=\frac{s}{s_{i}}\times \frac{c_{i}}{m}$$

where C is the concentration of the target compound (μg/g); c_i_ is the concentration of the internal standard; s is the peak area of the target compound; s_i_ is the peak area of the internal standard; and m is the sample mass (g).

(2) $$OAV=\frac{c_{i}}{{OT}_{i}}$$

where OAV is the odor activity value; c_i_ is the concentration of the compound in the sample; and OT_i_ is the sensory olfactory threshold of the compound in water [27].

(3) $$ACP=\frac{c_{i}}{c_{t}}$$

where ACP is the aroma characteristic proportion (%); c_i_ is the concentration of the compounds in the current characteristic set in the sample (μg/g); and c_t_ is the total concentration of all 19 compounds in the current sample (μg/g). The compounds included alpha-ionone, (E,E)-2,6-nonadienal, (E)-2-decenal, acetophenone, 2-ethyl-3,5-dimethylpyrazine, 2-methylfuran, dimethyl disulfide, dimethyl trisulfide, methional, 2-acetyl-1-pyrroline, pentanal, 1,3-dimethoxybenzene, benzaldehyde, (E)-2-heptenal, hexanal, 2-ethyl-1-hexanol, (E)-2-nonenal, (E,E)-2,4-decadienal, and 2-undecenal. Standards were purchased from the Sigma-Aldrich Chemical Company (St. Louis, MO, USA).

## 3. Results and Discussion

### 3.1. Differences in the Nutritional Composition of Potato-Based Reconstituted Rice Produced from Different Varieties

#### 3.1.1. Main Mineral Elements and Amino Acids

In terms of the amino acid composition (Table 1), significant differences in various taste-active amino acids, including tyrosine, isoleucine, valine, arginine, glycine, methionine, glutamic acid, and aspartic acid, were observed among the three types of reconstituted rice. Among these amino acids, isoleucine and methionine are essential amino acids for humans. No significant difference in the isoleucine content was observed between YPRR and RBRR. The methionine content in PPRR was 0.081 g/100 g, which was significantly higher than that in RBRR and YPRR (*p* < 0.05), indicating that PBRR has a similar or superior amino acid nutritional composition to RBRR. Glycine, glutamic acid, and aspartic acid are typical taste-active amino acids. The aspartic acid (0.76 g/100 g) and glutamic acid (1.38 g/100 g) contents in YPRR were significantly higher than those in RBRR and PPRR (*p* < 0.05), while the glycine content was slightly lower than that in RBRR (0.25 g/100 g). Umami is one of the main sensory characteristics of potatoes. The above results suggest that PBRR may have the unique umami characteristics of potatoes but is less sweet than RBRR. Therefore, if better taste is the goal, yellow potatoes are more suitable than purple potatoes for reconstituted rice production.

Minerals such as calcium and potassium are essential micronutrients for humans. Potassium intake is closely related to blood pressure regulation, cardiovascular health, and bone health [28]. The potassium content in PBRR is five times that of RBRR. Therefore, PBRR, as a high-potassium food, may help meet the daily potassium requirements. People usually supplement calcium through dairy products. The calcium content in PBRR is 1.1–1.7 times that of RBRR. As a plant-based food, PBRR may become an option as a calcium supplement for lactose-intolerant individuals [29]. Compared to other nutrients, iron is easily lost during processing due to interactions [30]. Notably, the iron content in YPRR and PPRR was 4.24 mg/100 g and 4.62 mg/100 g, respectively, which was 11–12 times that of the control group. The iron content in the human body is about 3–4 g, and it is mainly used for hemoglobin synthesis. As the most abundant trace element in the body, the iron demand is higher in lactating women [31]. Therefore, PBRR is particularly suitable for daily consumption by this population. Meta et al. [7] developed reconstituted rice with an iron content reaching 40 ppm using moringa. However, in China, moringa is a non-staple tropical crop, limiting its potential application in the large-scale production of low-cost iron-fortified rice substitutes. In contrast, this study selected potatoes as the main raw material, focusing more on the economic feasibility and wide availability of the raw materials. The high micronutrient characteristics of YPRR and PPRR may be related to the rich mineral content in potatoes themselves. Additionally, compared to the flour processing method that requires decolorization treatment, reconstituted rice made from fresh potatoes better retains trace elements during the extrusion process. In the future, high-potassium or high-iron potato varieties can be selected to further increase the mineral content of reconstituted rice and improve its application potential.

#### 3.1.2. Main Nutrients

Table 2 presents the main nutritional components of reconstituted rice produced from different potato varieties and compares them with RBRR in terms of energy and major nutritional functional characteristics. Starch and protein are key nutritional components affecting rice texture, and their ratio determines the mouthfeel of the final product. In this experiment, the starch contents of YPRR, PPRR, and RBRR were 74.4%, 71.1%, and 74.5%, respectively, with no significant differences among them. No significant differences in nutritional indicators such as the total protein content and total amino acid content were observed, indicating that PBRR is comparable to RBRR in terms of the energy supply. The reducing sugar contents of both PBRR types were significantly higher than that of RBRR. The reducing sugar content of YPRR was 2 g/100 g, which was significantly higher than that of PPRR (0.53 g/100 g) and RBRR (0.28 g/100 g) (*p* < 0.05). Reducing sugar components such as glucose can impart sweetness to the product and serve as important substrates for the Maillard reaction, promoting the formation of aroma compounds [32]. Compared to other potato products (such as French fries), reconstituted rice has lower processing temperatures during extrusion (below 150 °C), resulting in a lower risk of acrylamide formation. Additionally, this process has lower requirements for potato shape, allowing the effective utilization of irregular or small-sized potatoes and thus reducing the raw material requirements. Furthermore, since this process avoids high-temperature frying and other processing steps that readily produce acrylamide, potato varieties with higher reducing sugar contents also have application potential, thereby expanding the range of usable raw materials.

Dietary fiber is an important nutrient, and studies have confirmed that its intake is associated with reduced risks of various chronic diseases [33]. Compared to RBRR, dietary fiber was detected in both YPRR and PPRR, with contents of 3.92% and 3.44%, respectively, while traditional refined white rice typically contains only about 1% dietary fiber. Increasing the dietary fiber content in reconstituted rice has become important in food processing [34]. Liu et al. [35] successfully increased the dietary fiber content in reconstituted rice by adding soybean powder and proposed that soybean residue powder or rice bran could be further utilized to achieve this goal, but these raw materials may introduce undesirable flavors. Therefore, using fresh potatoes as raw material to increase the dietary fiber content in reconstituted rice is an ideal solution. Carotene is an important fat-soluble pigment and a precursor for vitamin A synthesis. Plant-based foods are important sources of vitamin A, playing crucial roles in maintaining vision, immunity, and skin health [36]. The carotene content in YPRR was 14.1 μg/g. Studies have shown that rice rich in β-carotene can alleviate vitamin A deficiency and iron deficiency anemia in populations consuming rice as a staple food [36]. The carotene content in YPRR is approximately half that of genetically modified golden rice (35 μg/g), but it has higher biosafety. Anthocyanins are potent natural antioxidants commonly found in dark-colored plants. The unique value of purple potatoes lies in their high contents of anthocyanins and polyphenolic compounds [37]. This study shows that the anthocyanin content in reconstituted rice made from purple potatoes can reach 45 mg/100 g. Consuming 100 g can meet the recommended intake in Chinese dietary guidelines, providing a feasible strategy for meeting the daily dietary needs. The anthocyanin content in the raw material variety ‘Diancaishu 101’ was 47 mg/100 g, indicating that reconstituted rice processed using fresh potatoes has good retention of easily degradable components such as anthocyanins. Xie et al. found that the soluble dietary fiber in purple potatoes exhibited a stronger free radical scavenging ability, suggesting possible synergistic effects between anthocyanins and dietary fiber [37]. Zhang et al. [38] increased the anthocyanin content in reconstituted rice by mixing indica rice with blueberry residue, and its antioxidant capacity was also significantly increased. Therefore, YPRR may also possess a greater antioxidant capacity.

In summary, PBRR is comparable to RBRR in terms of the energy supply while retaining the key nutritional components unique to different potato varieties. Therefore, yellow- or purple-fleshed potatoes can be selected for reconstituted rice production based on the nutritional needs of specific populations. Additionally, Bough et al. [26] found that potatoes with different flesh colors showed significant differences in sensory characteristics and volatile components due to differences in the contents of secondary metabolites such as carotenes and anthocyanins PBRR completely retains the plant pigments from fresh potatoes, which may also lead to differences in aroma formation pathways. Therefore, we further analyzed specific aroma compounds.

### 3.2. Flavor Characteristics of Potato-Based Rice Produced from Different Potatoes

#### 3.2.1. Changes in the Appearance of the Material and Quantitative Results for Main Volatile Compounds

**Figure 2:**
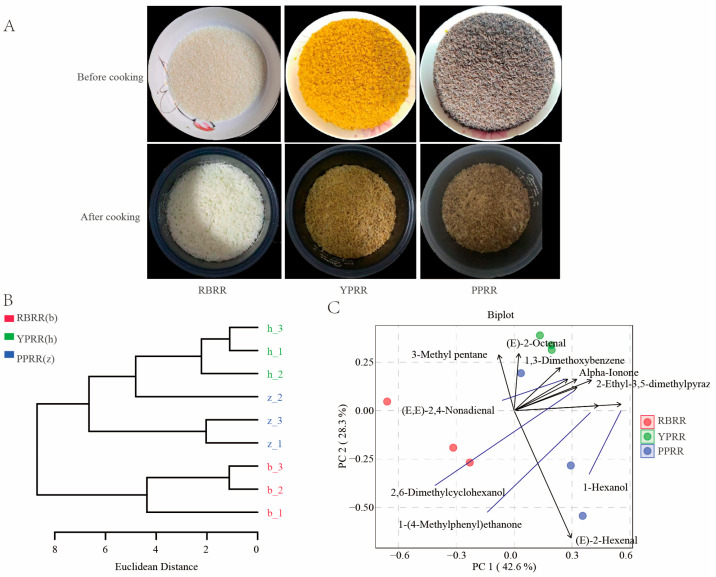
Appearance and evaluation of samples of reconstituted rice prepared using different materials. (**A**) Appearance of reconstituted rice (before and after cooking). (**B**) Results of the hierarchical clustering analysis of volatile compounds in reconstituted rice prepared using different materials. (**C**) Principal component analysis of reconstituted rice prepared using different materials. Note: Samples of the same color represent three replicates of the same material.

PBRR is a processing method that meets the needs of Chinese staple food consumption. It can be cooked in a rice cooker within 15 min because the PBRR has already been gelatinized during processing, thus achieving the long-term storage capability that fresh potatoes lack. Reconstituted rice typically retains the color of the raw material itself (Figure 2A). For example, plant leaves impart a green appearance to reconstituted rice [39,40]. After cooking, the appearance retains the color inherent to the potato because fresh potatoes are directly processed into reconstituted rice, eliminating the intermediate step of processing into flour and thus avoiding the loss of anthocyanins and carotenoids while preserving the characteristics of potato raw materials with reduced energy consumption. Like RBRR, macromolecules such as starch undergo denaturation during processing. PBRR resembles RBRR in texture aspects such as shape, size, and elasticity, making it more suitable for Chinese staple food consumption habits compared to the soft texture of directly steamed potato. The contents of aroma compounds differed between PBRR and RBRR, resulting in clear clustering separation between RBRR and PBRR (Figure 2B). Among PBRRs, differences also existed due to the use of different raw materials (Figure 2C). Principal component 1 explained 42.6% of the compositional variance, effectively distinguishing between PBRR and RBRR. The biplot shows that 1-hexanol, 1-(4-methylphenyl) ethanone, and 2-ethyl-3,5-dimethylpyrazine contributed significantly to the differentiation between PBRR and RBRR. PPRR and YPRR were distinguished along the PC2 direction, with differences in the contents of monounsaturated aldehydes such as (E)-2-hexenal and (E)-2-octenal between the two types of PBRR.

**Figure 3:**
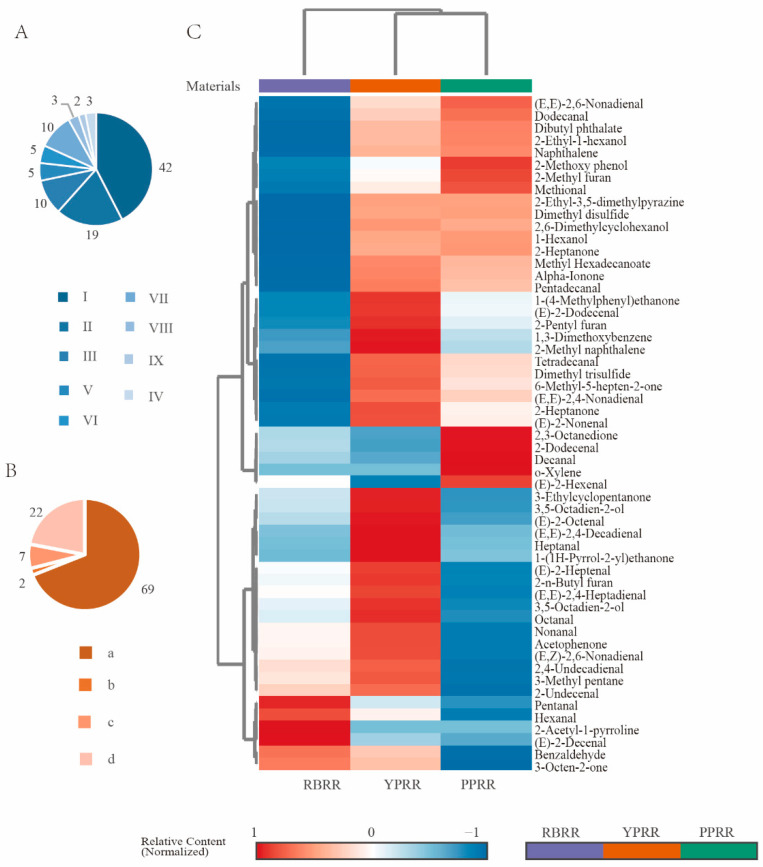
Quantitative results and classification of volatile compounds in reconstituted rice prepared using different materials. (**A**) Proportions of volatile compounds with different functional groups. (**B**) Proportions of volatile compounds from different sources. (**C**) Heatmap of the contents of major volatile compounds in three replicates of reconstituted rice samples prepared using different materials. The content of each compound is normalized to indicate the relative abundance, with red indicating a high content and blue indicating a low content. Note: In the figure, a indicates possible lipid degradation products, b indicates Maillard reaction (non-sulfur amino acid degradation) products, c indicates sulfur-containing amino acid degradation products, d indicates other sources; I represents aldehydes, II represents ketones, III represents alcohols, IV represents acids and esters, V represents sulfur-containing compounds, VI represents furans, VII represents aromatic hydrocarbons, VIII represents nitrogen-containing compounds, and IX represents phenols.

A total of 55 volatile compounds were detected in the experiment (Figure 3). After classification by aroma precursor sources (Figure 3A), lipid oxidation products accounted for the highest proportion (69%), followed by compounds from other sources (22%), while Maillard reaction and Strecker degradation products had the lowest proportion (9%). From the perspective of the chemical structure (Figure 3B), aldehydes had the highest relative content (42%), followed by ketones (19%) and alcohols (10%). After determining the contents of major volatile compounds in reconstituted rice prepared using different materials (Figure 3C), higher aldehydes and ketones contents were detected in YPRR than in PPRR and RBRR. This result is partly due to the higher contents of polyunsaturated fatty acids (such as linoleic acid) in potatoes, which are more prone to oxidation [41]. On the other hand, the abundant carotenoids in YPRR serve as precursors, and their thermal degradation also contributes to aldehydes and ketones, collectively increasing the overall content of these compounds. Notably, the 22% of compounds from “other sources” may originate from thermal degradation products of characteristic pigments (anthocyanins/carotenoids) in PPRR and YPRR, providing potential special aroma dimensions for PBRR. Furthermore, the low proportion of Maillard reaction products suggests a synergistic inhibitory effect between processing and raw materials: the high-moisture cooking conditions are unfavorable for this reaction, and the selected potato varieties may have low reducing sugar contents, limiting the Maillard pathway at the substrate level. Additionally, the slow heating process favors lipid oxidation-dominated flavor formation.

#### 3.2.2. Results of the Screen of Key Compounds Using Orthogonal Partial Least Squares and Correlation Methods

**Figure 4:**
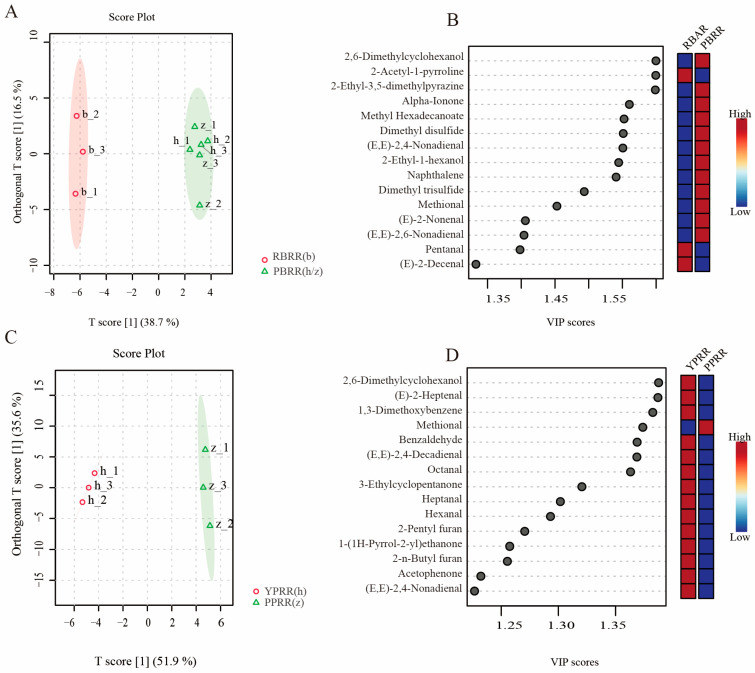
Discriminant analysis of differentially abundant aroma compounds between potato-based reconstituted rice and rice-based reconstituted rice, and between potato-based reconstituted rice prepared using different potato varieties. (**A**) Score plot of potato-based reconstituted rice and rice-based reconstituted rice. (**B**) VIP values of major compounds in the comparison between potato-based reconstituted rice and rice-based reconstituted rice. (**C**) Score plot of the reconstituted rice prepared using two potato varieties. (**D**) VIP values of the major compounds in the comparison between reconstituted rice prepared using different potato varieties. In the heatmap, the content of each compound represents the mean of three replicates normalized to indicate the relative abundance, with red indicating a high content and blue indicating a low content.

To further identify aroma compounds that distinguish PBRR and RBRR, orthogonal partial least squares discriminant analysis was performed on the two types of rice (Figure 4A). The results showed a T score of 38.7%, indicating certain inter-group differences, with samples within groups being relatively clustered, thus showing good representativeness. Further extraction of the 15 compounds with the highest VIP values (Figure 4B) showed that PPRR had higher contents of most compounds, such as 2-ethyl-3,5-dimethylpyrazine, 2,6-dimethylcyclohexanol, methional, dimethyl trisulfide, and dimethyl disulfide, which are nitrogen- and sulfur-containing compounds. Studies have suggested that 2-ethyl-3,5-dimethylpyrazine is a major source of the aroma of baked potatoes, with its content related to baking with the skin [42]. Additionally, polyunsaturated aldehydes and ketone compounds also showed higher levels of accumulation in potato-based reconstituted rice. In contrast, monounsaturated aldehyde compounds such as 2-acetyl-1-pyrroline, pentanal, and (E)-2-decenal accumulated in RBRR. Potatoes have a higher protein content than rice; therefore, their thermal degradation during processing leads to the formation of nitrogen-containing pyrazine compounds and Strecker degradation products such as sulfur-containing compounds. Meanwhile, substances such as carotenoids in PPRR further contribute to the production of ketone compounds [43]. 2-Acetyl-1-pyrroline is the main aroma compound in rice [44], and the difference in its content along with methional in reconstituted rice reflects the significant characteristics of the raw materials and can be used as characteristic flavor substances to authenticate PPRR.

Since nutrient distribution in raw materials can affect the aroma compound composition, we further conducted a differential analysis between PPRR and YPRR (Figure 4C). The T score value of 51% indicates significant differences in the aroma compound composition between the two types of PBRR. Further analysis revealed (Figure 4D) higher contents of saturated aldehydes represented by heptanal and hexanal, as well as polyunsaturated aldehydes represented by (E,E)-2,4-nonadienal and (E,E)-2,4-decadienal, in YPRR. Except for methional, other ketone compounds and furan compounds also showed greater accumulation in YPRR. Most aldehyde compounds are produced by fatty acid oxidation [45]. However, the difference in the aldehyde compound content between the two colored potato-based reconstituted rice varieties may be due to the abundance of antioxidants such as anthocyanins in PPRR, which inhibit the further oxidation of fatty acids, thus affecting the production of aldehydes from lipid degradation.

**Figure 5:**
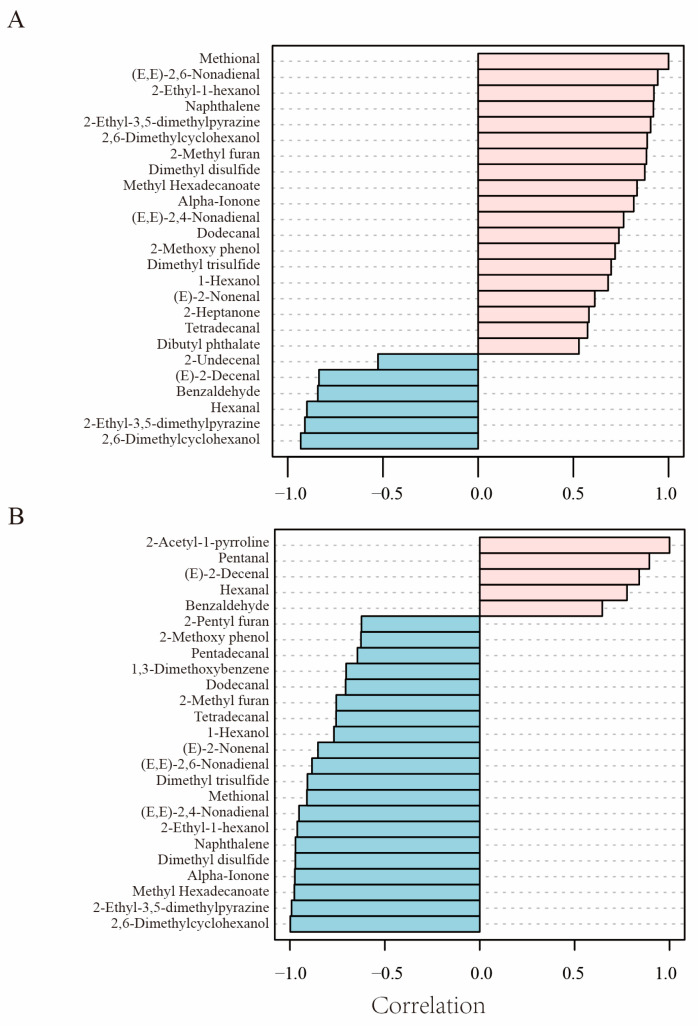
Pattern hunter analysis of key compounds. (**A**) Screening of aroma compounds correlated with the trends of changes in the methional content. (**B**) Screening of aroma compounds correlated with changes in the 2-acetyl-1-pyrroline content. The results of Spearman’s correlation analysis are shown, with red bars indicating positive correlations between the indicated compound and compounds in the first row, and blue bars indicating negative correlations between the indicated compound and compounds in the first row.

Methional and 2-acetyl-1-pyrroline are key aroma compounds in potatoes and rice, respectively, and the differences in raw material that they represent determine the differences in aroma compounds between potato-based reconstituted rice and RBRR. Therefore, compounds with similar or opposite content patterns to these two compounds are potential compounds that can reflect the characteristics of the aroma compound composition. Compounds most strongly correlated with the methional expression pattern (Figure 5A) include polyunsaturated aldehydes such as (E,E)-2,6-nonadienal, 2-ethyl-1-hexanol, 2-ethyl-3,5-dimethylpyrazine, nitrogen-containing compounds, sulfur-containing compounds, ketones, and alcohols. This result further confirms that PBRR has a high protein content and is rich in carotenoids and anthocyanins, leading to the inhibition of lipid oxidation and generation of abundant amino acid degradation products and pigment degradation products. In contrast, compounds with a content consistent with the pattern of 2-acetyl-1-pyrroline (Figure 5B) include degradation products such as pentanal, (E)-2-decenal, and benzaldehyde. Previous studies have suggested that fatty acids in rice are mainly monounsaturated fatty acids [44]; therefore, the degradation products are predominantly saturated aldehydes and monoenal aldehydes.

#### 3.2.3. Analyses of the OAVs and Aroma Characteristics of Different Varieties

Although many volatile compounds were detected, relatively few compounds play key roles in aroma formation [46]. Furthermore, using the criteria of VIP > 1 in OPLS-DA and FDR < 0.05 in the variance analysis, we screened 19 compounds among the 55 compounds as key differentially abundant compounds in YPRR, PPRR, and RBRR, and calculated odor activity values (OAVs) to explore the importance of these compounds in aroma quality formation [47]. As shown in Table 3, in RBRR, 2-acetyl-1-pyrroline had the highest activity value, followed by 2-ethyl-3,5-dimethylpyrazine, hexanal, benzaldehyde, and pentanal. These compounds impart a rice aroma, malty aroma, and toasted and fresh characteristics to RBRR [44]. In contrast, 2-ethyl-3,5-dimethylpyrazine had the highest activity value in PBRR, followed by methional, hexanal, α-ionone, pentanal, and (E, E)-2,4-decadienal. Therefore, based on the traditional malty aroma, it possesses characteristic potato aroma and fruity notes [48,49], resulting in an aroma composition with a more pronounced potato flavor. Compared to PPRR, methional contributed less in YPRR. While the characteristic potato odor was weaker, the fatty aroma represented by (E, E)-2,4-decadienal [50] and the fruity aroma represented by α-ionone were more prominent [49], along with a fresher aroma contributed by hexanal. Therefore, the overall flavor profile leans more toward traditional instant rice, balancing the characteristic potato flavor with traditional rice aroma characteristics and possessing the richest aroma qualities.

**Figure 6:**
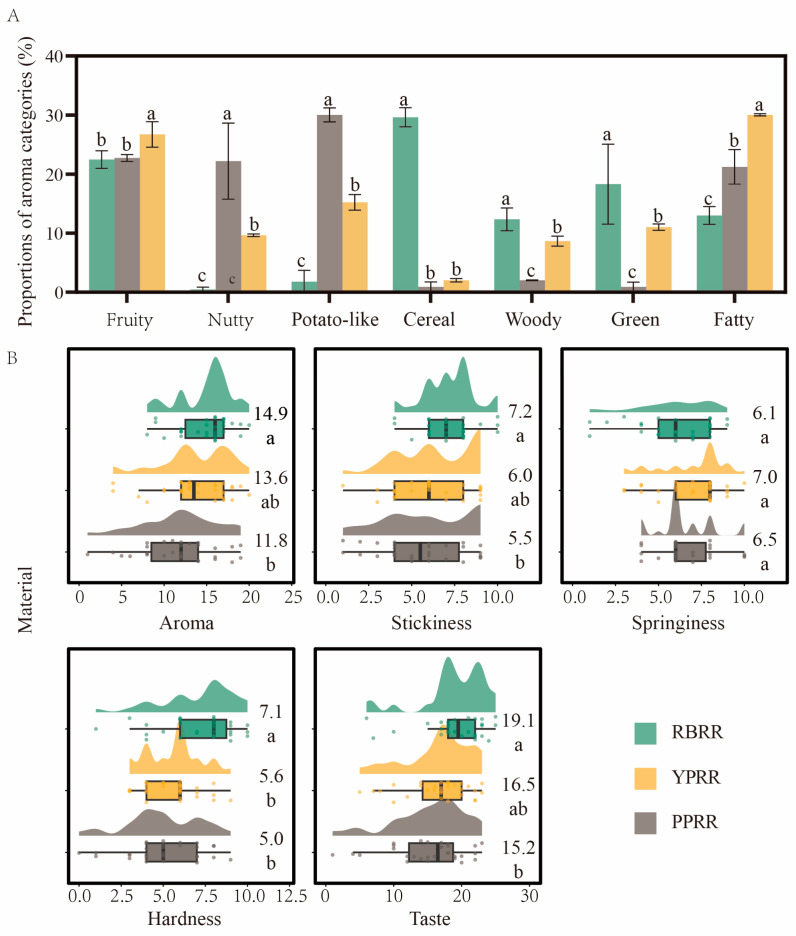
(**A**) Statistical results of the proportions of various aroma characteristic compounds in three types of reconstituted rice. (**B**) Statistics of the sensory evaluation scores for three types of reconstituted rice. With different superscript letters in the same row are significantly different (*p* < 0.05).

This study further clarifies the differences in aroma characteristics between PBRR and RBRR by systematically comparing the aroma attributes and relative contents of key aroma compounds (OAV > 1) in both samples. As shown in Figure 6A, the characteristic proportions of “potato-like” flavor (15.2%, 30%), “nutty” aroma (9.6%, 22%), and “fatty” aroma (30%, 21%) in YPRR and PPRR were significantly higher than those in RBRR (1.7%, 0.4%, 13%, respectively) (*p* < 0.05). PPRR and YPRR showed significant differences in their aroma compositions, with YPRR exhibiting higher “fruity” (26.7%) and “fatty” (30%) aroma characteristics and lower proportions of “nutty” aroma (9.6%) and “potato-like” flavor (15.2%). This phenomenon is consistent with genetic studies on potato flavor, which indicate that differences in the contents of precursors such as amino acids, sugars, and lipids between varieties directly determine the composition and relative proportions of volatile aroma compounds [49]. Thybo et al. [51] found that the intensity of the “potato-like” flavor and “fatty” flavor differed significantly among potato varieties, and these differences were mainly attributed to the variety. Morris et al. [52] also noted that *Phureja* varieties exhibited more pronounced umami and intense flavors than Tuberosum varieties, which were closely related to their higher glutamate levels. Furthermore, variety-related differences in flavor characteristic proportions are prevalent in food products, fundamentally due to the diversity in the raw material’s chemical composition and differences in reaction pathways occurring during processing. For example, different protein sources (such as pea versus soybean) vary in the sulfur-containing amino acid content, thereby affecting the proportions of “meaty” and “beany” aromas in the overall profile [53]. Similarly, tomato varieties with a higher carotenoid content generate more terpenoids such as ionones through oxidative cleavage, significantly increasing the proportion of “fruity” characteristics in the product [54]. These results also demonstrate that the aroma differences between different colored potato-based reconstituted rice are closely related to their variety-specific metabolite composition: higher carotenoid precursor contents in YPRR are more readily degraded to generate “fruity” aroma components. Compared to RBRR, PBRR contains more sulfur-containing precursors (such as methionine), and the sulfur-derived flavor compounds (such as dimethyl trisulfide) produced after thermal processing significantly enhance the characteristic “potato-like” aroma. Additionally, higher levels of taste-active amino acids such as glutamate in potatoes may synergistically enhance “umami” and “nutty” flavors. Therefore, achieving targeted flavor regulation by breeding varieties with specific metabolic characteristics (such as high-methionine or high-carotenoid genotypes) is a feasible strategy.

Sensory evaluation is a direct factor in measuring product quality, and the goal of potato-based reconstituted rice is to achieve nutritional enhancement over traditional cereal-based reconstituted rice. Therefore, the sensory evaluation standards were based on the Chinese national standard sensory evaluation for traditional rice (Figure 6B). Overall, certain differences in sensory characteristics were observed between PBRR and RBRR. The sensory evaluation results showed that YPRR scored significantly lower than RBRR (*p* < 0.05) in four indicators: aroma (11.8), taste (15.2), stickiness (5.5), and hardness (5.0). When yellow potatoes were used as the raw material, the product’s scores for aroma (13.6), taste (16.5), and stickiness (6.0) all improved, with no significant differences from RBRR.

The analysis of volatile compounds showed that potato-based reconstituted rice had a higher proportion of “potato-like” characteristic aroma components, while “cereal” aroma components were relatively lacking. This difference in aroma composition directly affected the sensory evaluation results. Notably, the aroma scores for yellow potato-based rice showed a bimodal distribution, indicating that evaluation panel members had divergent acceptance of the characteristic potato aroma. This phenomenon is consistent with the findings of Zhao et al. [55], who found that when the methional content in cooked potatoes was excessively high and lacked balance from other aroma compounds, it would produce off-flavors. In this study, some evaluators perceived the prominent characteristic potato aroma as an “off-flavor” deviating from the traditional rice aroma, leading to reduced acceptance; meanwhile, another group of evaluators considered this unique aroma between rice and potato to have positive sensory characteristics. This difference in evaluation indicates that the intensity and compositional ratio of characteristic potato aromas are key factors affecting the sensory acceptance of the product.

Regarding springiness, most evaluators considered yellow potato-based rice to be springier, which may be related to its starch depletion pattern. During the processing of reconstituted rice, starch granules undergo gelatinization and recrystallization processes, leading to changes in the original starch structure [9]. Compared to rice starch, potato starch has a larger granule size and more complex granule morphology [56], which may result in a lower degree of starch depletion during heat treatment, avoiding insufficient gelatinization and excessive degradation and thereby imparting better springiness properties to the product [57]. However, it showed no statistically significant difference from RBRR, potentially due to textural heterogeneity caused by localized starch depletion, leading some evaluators to perceive relatively poorer springiness performance. The extrusion parameters of reconstituted rice (such as temperature and the moisture content) significantly affect the microstructure and hydration behavior of reconstituted rice [58]. In the future, by optimizing the processing parameters, the degree of starch depletion can be further controlled for different potato variety raw materials to achieve a more stable springiness texture.

Sensory quality issues in reconstituted rice are prevalent throughout the industry. Due to fundamental differences between reconstituted rice and natural rice in the volatile compound composition, amylose-to-amylopectin ratio, and protein and carbohydrate contents, meeting consumers’ inherent expectations for the sensory characteristics of traditional rice such as aroma, texture, and taste is difficult. Multiple studies have confirmed this phenomenon. Jiang et al. [13] found that the appearance, palatability, taste, cold rice texture, and overall scores of *Pleurotus eryngii* extruded rice all decreased with increasing amounts of *Pleurotus eryngii* powder. Zhang et al. [9] noted that even nutritionally fortified rice containing millet, corn, and other cereals still had considerable room for improvement in eating quality. Mahendradatta et al. [7] also showed that nutritional rice prepared from sago, corn flour, and moringa leaf powder exhibited a trend of decreasing sensory scores with increasing moringa leaf addition. Traditional improvement strategies mainly rely on adjusting the raw material ratios, but this approach often sacrifices the nutritional design objectives of the product. Our study found that selecting different potato varieties can significantly improve the sensory performance of products while maintaining unchanged nutritional ratios. This optimization strategy based on variety selection provides a more flexible solution for improving the sensory quality of reconstituted rice.

Overall, PBRR made from different colored potatoes can provide a flavor experience distinct from traditional rice. Variations in raw materials and blending ratios can enhance the sensory experience [59]. Therefore, future efforts could explore mixing and adjusting the proportions of different colored potato varieties to expand product flavor profiles. However, PBRR currently has flavor shortcomings such as a weak “cereal” aroma. We recommend that future work combine a detailed sensory evaluation with processing optimization (such as adjusting the thermal processing conditions to balance the Maillard reaction and lipid oxidation) to specifically strengthen the aroma structure and improve acceptance of the product. Based on the current results, we recommend that consumers who prefer a typical “potato-like” flavor choose PPRR, while those more accustomed to a traditional rice style may find YPRR more suitable.

## 4. Conclusions

Potato-based reconstituted rice (PBRR) is a semi-cooked instant food produced through the refined processing of potatoes, representing a new approach to potato deep processing. Due to its flexible texture and high nutritional value, PBRR has become a potential product for increasing potato consumption choices. Consumers perceive its flavor as unique, combining the aroma of traditional rice with potato characteristics, yet research into its specific aroma compound composition is lacking. In this study, using rice-based reconstituted rice (RBRR) as a control, we quantitatively analyzed the aroma compounds of purple and yellow potato-based reconstituted rice using HS-SPME-GC-MS technology. The results showed significant differences in the aroma compound composition between PBRR and RBRR. The detected aroma compounds were mainly aldehydes, ketones, and alcohols, with lipid oxidation degradation products accounting for more than half of all detected compounds. Through OPLS-DA, methional, 2-ethyl-3,5-dimethylpyrazine, α-ionone, and (E, E)-2,6-nonadienal were identified as differentially abundant compounds distinguishing PBRR from RBRR. These compounds impart the characteristic “potato-like” aroma, as well as “nutty”, “fruity”, and “fatty” flavors to PBRR. Therefore, consumers with a preference for the potato flavor can choose purple potato-based reconstituted rice to obtain a sensory experience closer to eating potatoes, while consumers more accustomed to traditional rice are recommended to choose yellow potato-based reconstituted rice for a richer flavor experience.

## Figures and Tables

**Table 1:** Statistics of the contents of the major mineral elements and amino acids in different reconstituted rice samples.

Compound	Reconstituted Rice		
	RBRR	YPRR	PPRR
Mineral elements (mg/100 g)			
Calcium	67.90 ± 3.65 b	114.00 ± 2.70 a	74.20 ± 7.02 b
Potassium	899.00 ± 79.36 b	4360.00 ± 109.86 a	4170.00 ± 134.78 a
Magnesium	197.00 ± 8.08 b	299.00 ± 8.16 a	287.00 ± 5.33 a
Iron	3.60 ± 0.15 b	42.40 ± 2.46 a	46.20 ± 4.53 a
Zinc	12.70 ± 0.87 a	7.12 ± 0.57 b	7.98 ± 0.37 b
Amino acids (g/100 g)			
Total amino acids	5.130 ± 0.330 a	5.640 ± 0.230 a	4.850 ± 0.090 a
Histidine	0.120 ± 0.001 a	0.130 ± 0.002 a	0.120 ± 0.007 a
Tyrosine	0.022 ± 0.002 b	N.D.	0.086 ± 0.006 a
Isoleucine	0.220 ± 0.009 a	0.210 ± 0.019 a	0.150 ± 0.003 b
Valine	0.320 ± 0.029 a	0.300 ± 0.009 ab	0.230 ± 0.003 b
Alanine	0.330 ± 0.013 a	0.380 ± 0.023 a	0.360 ± 0.023 a
Arginine	0.360 ± 0.025 a	0.220 ± 0.009 b	0.200 ± 0.008 b
Lysine	0.190 ± 0.018 a	0.210 ± 0.017 a	0.190 ± 0.013 a
Serine	0.290 ± 0.004 a	0.270 ± 0.013 a	0.250 ± 0.009 a
Glutamic acid	1.130 ± 0.052 b	1.380 ± 0.074 a	1.110 ± 0.011 b
Glycine	0.250 ± 0.007 a	0.200 ± 0.023 ab	0.190 ± 0.007 a
Methionine	0.069 ± 0.004 b	0.048 ± 0.001 c	0.081 ± 0.001 a
Leucine	0.540 ± 0.032 a	0.640 ± 0.007 a	0.560 ± 0.036 a
Phenylalanine	0.270 ± 0.006 a	0.270 ± 0.005 a	0.270 ± 0.015 a
Threonine	0.200 ± 0.016 a	0.200 ± 0.007 a	0.190 ± 0.006 a
Aspartic acid	0.530 ± 0.001 b	0.760 ± 0.048 a	0.500 ± 0.017 b

**Note:** Values are presented as means ± standard deviations. Data with different lowercase letters in the same row are significantly different (*p* < 0.05).

**Table 2:** Main nutrients in reconstituted rice produced from different potato varieties.

Compound	Reconstituted Rice		
	RBRR	YPRR	PPRR
Protein (g/100 g)	3.41 ± 1.40 a	3.63 ± 1.44 a	3.38 ± 1.35 a
Starch (g/100 g)	74.50 ± 5.60 a	74.40 ± 4.20 a	71.10 ± 1.90 a
Dry matter (g/100 g)	86.80 ± 2.70 a	88.00 ± 1.30 a	86.90 ± 2.90 a
Reducing sugars (g/100 g)	0.28 ± 0.01 c	2.00 ± 0.10 a	0.53 ± 0.01 b
Carotenoids (μg/100 g)	N.D.	14.10 ± 0.80 a	N.D.
Anthocyanins (mg/100 g)	N.D.	N.D.	45.00 ± 2.63 a
Dietary fiber (g/100 g)	N.D.	3.92 ± 0.13 a	3.44 ± 0.04 b

**Note:** Values are presented as means ± standard deviations. Data with different lowercase letters in the same row are significantly different (*p* < 0.05).

**Table 3:** OAV analysis of key volatile compounds.

Compound	Odor Threshold (mg/kg)	OAV			Aroma Description	Aroma Classification
		RBRR	PPRR	YPRR		
Alpha-Ionone	0.0001	80	650	930	Violet, berry	Fruity
(E,E)-2,6-Nonadienal	0.0011	4.54	33.63	15.45	Cucumber, green	Fruity
(E)-2-Decenal	0.003	2	0.33	0.66	Citrus peel, fatty	Fruity
Acetophenone	0.05	0.28	0.12	0.64	Orange blossom, cherry	Fruity
2-Ethyl-3,5-dimethylpyrazine	0.00001	2100	40,000	40,800	Roasted nut, roasted coffee	Nutty
2-Methyl furan	0.0014	17.85	132.85	45	Roasted coffee, caramel	Nutty
Dimethyl disulfide	0.009	0.91	3.16	3.06	Garlic, roasted potato	Potato-like
Dimethyl trisulfide	0.007	0.54	1.23	2.05	Cooked onion, roasted potato	Potato-like
Methional	0.00006	0.01	6158.73	1952.38	Boiled potato	Potato-like
2-Acetyl-1-pyrroline	0.00002	4300	0	0	Toasty, rice-like	Cereal
Pentanal	0.0014	630.71	302.85	385	Bread, malt	Cereal
1,3-Dimethoxybenzene	0.0241	0.083	0.16	1.03	Anise, herbal	Woody
Benzaldehyde	0.01	116.8	60.9	98.3	Cherry pit, roasted nut	Woody
(E)-2-Heptenal	0.011	5.36	3.72	7.455	Grassy, fatty	Green
Hexanal	0.0067	1772.53	835.97	1253.88	Grassy, unripe fruit	Green
2-Ethyl-1-hexanol	0.74	0.63	1.45	1.26	Floral, slight green	Fatty
(E)-2-Nonenal	0.0034	12.05	19.70	28.52	Grassy, fatty	Fatty
(E,E)-2,4-Decadienal	0.005	141.6	136.8	905.2	Fried, nutty	Fatty
2-Undecenal	0.04	1.17	0.35	2.02	Citrus, fatty	Fatty

## Data Availability

Data is contained within the article.

## References

[1] Wang Z., Liu H., Zeng F., Yang Y., Xu D., Zhao Y.-C., Liu X., Kaur L., Liu G., Singh J. (2023). Potato Processing Industry in China: Current Scenario, Future Trends and Global Impact. Potato Res..

[2] Saha S., Roy A. (2020). Whole Grain Rice Fortification as a Solution to Micronutrient Deficiency: Technologies and Need for More Viable Alternatives. Food Chem..

[3] Sumardiono S., Pudjihastuti I., Poerwoprajitno A.R., Suswadi M.S. (2014). Physichocemical Properties of Analog Rice from Composite Flour: Cassava, Green Bean and Hanjeli. World Appl. Sci. J..

[4] Lal M.K., Singh B., Sharma S., Singh M.P., Kumar A. (2021). Glycemic Index of Starchy Crops and Factors Affecting Its Digestibility: A Review. Trends Food Sci. Technol..

[5] Kumar A., Sahu C., Panda P.A., Biswal M., Sah R.P., Lal M.K., Baig M.J., Swain P., Behera L., Chattopadhyay K. (2020). Phytic Acid Content May Affect Starch Digestibility and Glycemic Index Value of Rice (*Oryza sativa* L.). J. Sci. Food Agric..

[6] Purwaningsih S., Santoso J., Handharyani E., Setiawati N.P., Deskawati E. (2020). Artificial Rice from *Gracillaria* Sp. as Functional Food to Prevent Diabetes. IOP Conf. Ser. Earth Environ. Sci..

[7] Mahendradatta M., Rombe T.E., Rahman A.N.F., Langkong J., Tawali A.B., Nadhifa D.G. (2024). Analog Rice Based on Sago and Corn with the Addition of Moringa Leaf (*Moringa oleifera* L.) Powder as a Nutritional Vehicle for Breastfeeding Women. Foods.

[8] Saha S., Jha S., Tiwari A., Jayapalan S., Roy A. (2021). Considerations for Improvising Fortified Extruded Rice Products. J. Food Sci..

[9] Zhang D., Li J., Yao D., Wu J., Luo Q., Shen H., Hu M., Meng F., Zhang Y., Liu X. (2024). Differences in Cooking Taste and Physicochemical Properties between Compound Nutritional Rice and Common Rice. Front. Nutr..

[10] Damat D., Setyobudi R.H., Burlakovs J., Vincevica-Gaile Z., Siskawardani D.D., Anggriani R., Tain A. (2021). Characterization Properties of Extruded Analog Rice Developed from Arrowroot Starch with Addition of Seaweed and Spices. Sarhad J. Agric..

[11] Nadhifa D.G., Mahendradatta M., Poespitasari A., Bastian F., Adhnitasari A.Y. (2025). Characterization of Analog Rice Produced from Various Carbohydrate Sources and Their Functional Components: A Review. Discov. Food.

[12] Ramadhan W., Purwaningsih S., Nafisah S. (2024). Effects of Microwave Cooking on the Physicochemical and Sensory Properties of Seaweed-based Analogue Rice. Int. J. Food Sci. Technol..

[13] Jiang W., Liang W.X., Pei F., Su A.X., Ma G.X., Fang D.L., Hu Q.H., Ma N. (2024). Effects of Adding *Pleurotus eryngii* Powder on Quality Characteristics of Extruded Rice. Sci. Agric. Sin..

[14] Olsson K., Svensson R., Roslund C. (2004). Tuber Components Affecting Acrylamide Formation and Colour in Fried Potato: Variation by Variety, Year, Storage Temperature and Storage Time. J. Sci. Food Agric..

[15] Zhang Q., Sun Y., Sun Y., Guo C., Zhu J., Niu X., Gao M. (2024). Comparative Analysis of Physicochemical Properties, Sensory Characteristics, and Volatile Flavor Compounds in Five Types of Potato Chips. Front. Nutr..

[16] (2016). Determination of Protein in Foods.

[17] (2023). Determination of Starch in Foods.

[18] (2016). Determination of Moisture in Foods.

[19] (2016). Determination of Carotene in Foods.

[20] (2016). Determination of Multi-Elements in Foods.

[21] (2016). Determination of Amino Acids in Foods.

[22] (2016). Determination of Reducing Sugar in Foods.

[23] (2023). Determination of Dietary Fiber in Foods.

[24] (2025). Determination of Total Anthocyanins in Plant-Origin Food Agricultural Products.

[25] (2008). Sensory Evaluation Method for Cooking and Eating Quality of Paddy Rice and Brown Rice.

[26] Bough R. (2017). Profiling and Putative Aroma Biomarker Identification for Flavor in Potatoes Using a Trained Sensory Panel and HS-SPME GC-MS. Master’s Thesis.

[27] Van Gemert L.J. (2011). Odour Thresholds: Compilations of Odour Threshold Values in Air, Water and Other Media.

[28] Farag M.A., Abib B., Qin Z., Ze X., Ali S.E. (2023). Dietary Macrominerals: Updated Review of Their Role and Orchestration in Human Nutrition throughout the Life Cycle with Sex Differences. Curr. Res. Food Sci..

[29] Antunes I., Bexiga R., Pinto C., Roseiro L., Quaresma M.A.G. (2023). Cow’s Milk in Human Nutrition and the Emergence of Plant-Based Milk Alternatives. Foods.

[30] Prentice A.M., Mendoza Y.A., Pereira D., Cerami C., Wegmuller R., Constable A., Spieldenner J. (2017). Dietary Strategies for Improving Iron Status: Balancing Safety and Efficacy. Nutr. Rev..

[31] Cai C., Liu Y., Xu Y., Zhang J., Wei B., Xu C., Wang H. (2025). Mineral-Element-Chelating Activity of Food-Derived Peptides: Influencing Factors and Enhancement Strategies. Crit. Rev. Food Sci. Nutr..

[32] Ajandouz E.H., Tchiakpe L.S., Ore F.D., Benajiba A., Puigserver A. (2001). Effects of pH on Caramelization and Maillard Reaction Kinetics in Fructose-Lysine Model Systems. J. Food Sci..

[33] Anderson J.W., Baird P., Davis R.H., Ferreri S., Knudtson M., Koraym A., Waters V., Williams C.L. (2009). Health Benefits of Dietary Fiber. Nutr. Rev..

[34] Tyl C., Bresciani A., Marti A. (2021). Recent Progress on Improving the Quality of Bran-Enriched Extruded Snacks. Foods.

[35] Liu X., Zhao J., Zhang X., Li Y., Zhao J., Li T., Zhou B., Yang H., Qiao L. (2018). Enrichment of Soybean Dietary Fiber and Protein Fortified Rice Grain by Dry Flour Extrusion Cooking: The Physicochemical, Pasting, Taste, Palatability, Cooking and Starch Digestibility Properties. RSC Adv..

[36] Tang G., Qin J., Dolnikowski G.G., Russell R.M., Grusak M.A. (2009). Golden Rice Is an Effective Source of Vitamin A. Am. J. Clin. Nutr..

[37] Xie F., Zhang W., Lan X., Gong S., Wu J., Wang Z. (2017). Physicochemical Properties and Structural Characteristics of Soluble Dietary Fibers from Yellow and Purple Fleshed Potatoes By-Product. Int. J. Food Prop..

[38] Zhang X., Gao Y., Wang R., Sun Y., Li X., Liang J. (2022). Effects of Adding Blueberry Residue Powder and Extrusion Processing on Nutritional Components, Antioxidant Activity and Volatile Organic Compounds of Indica Rice Flour. Biology.

[39] Sailendra N.V., Aji A.S., Saloko S., Aprilia V., Djidin R.T.S., Rahmawati S., Khoirunnisah F.M. (2024). The Effect of Rice Cooking Techniques on Sensory Evaluation and Stickiness Level of Rice from Analog Rice Made from Sorghum Flour, Mocaf, Glucomannan, and Moringa. Amerta Nutr..

[40] Mahendradatta M., Assa E., Langkong J., Tawali A.B., Nadhifa D.G. (2024). Development of Analog Rice Made from Cassava and Banana with the Addition of Katuk Leaf (*Sauropus androgynous* L. Merr.) and Soy Lecithin for Lactating Women. Foods.

[41] Raigond P., Singh B., Dutt S., Kumar Chakrabarti S. (2020). Potato: Nutrition and Food Security.

[42] Oruna-Concha M.J., Duckham S.C., Ames J.M. (2001). Comparison of Volatile Compounds Isolated from the Skin and Flesh of Four Potato Cultivars after Baking. J. Agric. Food Chem..

[43] Jansky S.H. (2010). Potato Flavor. Am. J. Potato Res..

[44] Hu X., Lu L., Guo Z., Zhu Z. (2020). Volatile Compounds, Affecting Factors and Evaluation Methods for Rice Aroma: A Review. Trends Food Sci. Technol..

[45] Zamora R., Gallardo E., Hidalgo F.J. (2007). Strecker Degradation of Phenylalanine Initiated by 2,4-Decadienal or Methyl 13-Oxooctadeca-9,11-Dienoate in Model Systems. J. Agric. Food Chem..

[46] Comandini P., Cerretani L., Blanda G., Bendini A., Toschi T.G. (2011). Characterization of Potato Flavours: An Overview of Volatile Profiles and Analytical Procedures. Potato V: Food 5 (Special Issue 1).

[47] Xiao D.-R., Liu R.-S., He L., Li H.-M., Tang Y.-L., Liang X.-H., Chen T., Tang Y.-J. (2015). Aroma Improvement by Repeated Freeze-Thaw Treatment during Tuber Melanosporum Fermentation. Sci. Rep..

[48] Blanda G., Cerretani L., Comandini P., Toschi T.G., Lercker G. (2010). Investigation of Off-Odour and off-Flavour Development in Boiled Potatoes. Food Chem..

[49] McKenzie M., Corrigan V., Singh J., Kaur L. (2016). Potato Flavor. Advances in Potato Chemistry and Technology.

[50] Dresow J.F., Böhm H. (2009). The Influence of Volatile Compounds of the Flavour of Raw, Boiled and Baked Potatoes: Impact of Agricultural Measures on the Volatile Components. Landbauforschung.

[51] Thybo A.K., Christiansen J., Kaack K., Petersen M.A. (2006). Effect of Cultivars, Wound Healing and Storage on Sensory Quality and Chemical Components in Pre-Peeled Potatoes. LWT Food Sci. Technol..

[52] Morris W.L., Shepherd T., Verrall S.R., McNicol J.W., Taylor M.A. (2010). Relationships between Volatile and Non-Volatile Metabolites and Attributes of Processed Potato Flavour. Phytochemistry.

[53] Schutte L., Teranishi R. (1974). Precursors of Sulfur-containing Flavor Compounds. CRC Crit. Rev. Food Technol..

[54] Simkin A.J., Schwartz S.H., Auldridge M., Taylor M.G., Klee H.J. (2004). The Tomato Carotenoid Cleavage Dioxygenase 1 Genes Contribute to the Formation of the Flavor Volatiles β-Ionone, Pseudoionone, and Geranylacetone. Plant J..

[55] Zhao B., Zhang M., Liang S. (2017). Effect of overcooking on flavor compounds of potato. Food Sci..

[56] Guo L., Chen H., Zhang Y., Yan S., Chen X., Gao X. (2023). Starch granules and their size distribution in wheat: Biosynthesis, physicochemical properties and their effect on flour-based food systems. Comput. Struct. Biotechnol. J..

[57] Huang X., Liu H., Ma Y., Mai S., Li C. (2022). Effects of extrusion on starch molecular degradation, order–disorder structural transition and digestibility—A review. Foods.

[58] Mishra A., Mishra H.N., Srinivasa Rao P. (2012). Preparation of rice analogues using extrusion technology. Int. J. Food Sci. Technol..

[59] Kusmiandany E., Pratama Y., Pramono Y.B. (2019). The Effect of Gatot (Fermented Dried Cassava) and Red Bean Ratio on Water Content and Organoleptic Characteristics of the “Gatotkaca” Analog Rice. J. Appl. Food Technol..

